# Older people’s day centres’ preventive work: views of day centre providers and their stakeholders

**DOI:** 10.1080/17482631.2025.2500852

**Published:** 2025-05-13

**Authors:** Kritika Samsi, Katharine Orellana

**Affiliations:** NIHR Policy Research Unit in Health & Social Care Workforce, King’s College London, London, UK

**Keywords:** Day centre, day care, prevention, anticipatory care, older people

## Abstract

**Purpose:**

Day centres in England provide important social support to people living in the community. Our study aimed to further understandings of day centres’ contribution to health and social care’s preventive agenda.

**Methods:**

After obtaining ethical approval, we conducted qualitative interviews with 10-day centre stakeholders and 9 professional stakeholders, exploring their perceptions of day centres’ preventive function. Participants’ job roles provide context, while individual characteristics are anonymized.

**Results:**

Thematic analysis identified that day centre staff and volunteer activities to monitor attenders and intervene when needed align with the primary, secondary and tertiary prevention framework. A fourth theme, systemic opportunities, incorporates evidence, joint working and challenges.

**Conclusions:**

Day centres for older people can be well-placed to contribute to integrated care’s prevailing preventive agenda. However, tight budgets and limited joint commissioning practices potentially miss the benefits of anticipatory care day centres may offer to maintaining well-being and preventing deterioration.

## Introduction

Social care and healthcare demands are inextricably linked. Financial constraints and rationing, through needs-related eligibility criteria and means-testing, have contributed to declining rates of people accessing long-term support from local authority (LA) social care services, despite increased requests; even carer support and short-term reablement packages have fallen Hoddinott et al. (2023). Unmet social care needs will likely increase demand for healthcare services, particularly from general practitioners (GPs, family doctors) and Accident and Emergency (A&E). Although the potential impact is difficult to quantify, we know that social care funding cuts lead to, for example, more A&E visits; the average cut of £375 per-person adult social spending between 2009/10 and 2015/16 led to a 24.3% increase in A&E attendance (Crawford et al., 2021).

Prevention is a goal firmly embedded in England’s health and social care policy. Within social care policy, prevention—actively promoting independence and wellbeing and intervening early to prevent or delay the onset of further needs or decline—is one of LAs’ key responsibilities (HM Government, 2014, 2016). Within the NHS, primary prevention (promoting wellbeing) aims to help prevent disease or the need for care and support on a population-wide level; secondary prevention (early intervention) focuses on slowing or preventing further needs from developing in the early stages of disease or with people at risk, and tertiary prevention focuses on delaying or minimizing the impact of complex or multiple health conditions (maintenance). Reducing current and future state expenditure is the main driver behind preventive activity (Verity et al., 2022). Current preventive efforts aim to reduce pressure on the NHS and to move more care upstream, into the community (NHS, 2019). Key to this is proactive, anticipatory care—undertaken jointly between all parts of the health system and the voluntary and social care sectors—that supports people with complex needs or at risk of poor health outcomes to age well, reduces the need for reactive health care and addresses the wider determinants of health (NHS, 2019). Some NHS leaders feel that partnership working to improve primary and secondary prevention work could be (further) developed, despite funding challenges (Faculty of Public Health, 2019).

Despite repeated government focus on growing community health and care services, the failure to invest in these has been noted, with the various contributing factors including short-term approaches to investment, urgent problems trumping a focus on longer-term preventive efforts, the need for cost-saving and a “cycle of invisibility” among community services (Baird et al., 2024). Furthermore, enactment of prevention within social care has been undermined by a lack of consensus on the concept and local strategies being influenced by available budget rather than policy (Marczak et al., 2019).

The day centre for older people is an interesting example of an often invisible preventive social care service, cited in guidance supporting LAs to make a strategic shift to prevention and early intervention (Robertson & with support from Kaur, R., Glew, A., Marcelgelo, N. and the 29 POPPs sites, 2008). Day centres are community building-based services providing care/health-related services and/or activities specifically for people who are disabled/in need, that can be attended (usually on a pr-planned basis) for a whole or part of a day, and that support people to remain living at home and enable carers to sustain care (Orellana et al., 2024). Services operate in the statutory, voluntary, and private sectors. Many people regularly attending day centres may have multiple long-term conditions, be aged over 80, live alone and struggle to go out of their homes without support (Lunt et al., 2021; Orellana et al., 2020a). This profile places them within the National Institute for Health and Care Excellence’s (NICE) category of “vulnerable older people” who are “most at risk of a decline in their independence and mental wellbeing” (National Institute for Health and Care Excellence, 2015:para 1.5.3).

Given day centre attenders’ profile and centres’ preventive aims (Orellana et al., 2020b, 2023), how these services support the preventive agenda of the health and social care sector more formally is of interest. Recent empirical research about English day centres reports preventive monitoring, with necessary follow-ups, being undertaken at them (Orellana et al., 2020a, 2023), attendance resulting in a reduction in levels of anxiety and depression and an improvement in health and wellbeing during the first 12 weeks, regardless of whether staffed by paid staff or volunteers (Lunt et al., 2021) and day centres hosting NHS falls prevention exercise classes (Orellana et al., 2023). Bennett et al. (2023) and the afore-mentioned study authors highlight the potential for what day centres (particularly services for older people without the highest physical and cognitive needs) do and could offer within the context of the preventive and early intervention approach promoted by health and social care policy, and highlight how partnerships with health can enhance their preventative role.

Central to improved collaborative work and further investment in day centres is a better understanding of stakeholders’ perceptions of them and their preventive role. However, there is scant research about the views of health and social care professionals who are often in contact with older people needing care and support (e.g., family doctors (GPs), nurses, occupational therapists, social prescribers, social workers). One English study, which explored a small number of LA staff views of day centres’ relevance to social care policy themes, reported that social care practitioners and commissioners believed that services promoted wellbeing and prevented or delayed deterioration. Nevertheless, commissioners did not always link these outcomes with these services addressing the related policy goal (Orellana, 2018).

## Methods

### Study aim, design and setting

This article reports qualitative, semi-structured interviews with day centre providers and stakeholders for their perceptions of day centres’ role and contribution to the prevention priorities of the health and care sectors. This topic was explored within a larger study developing a set of day centre-related resources for day centres for older people and their stakeholders (see https://arc-sl.nihr.ac.uk/day-centre-resources-hub) following a survey that identified a lack of supportive resources and revealed an appetite for joint working (Orellana et al., 2024). We define day centre “stakeholders” as people with a personal or professional interest, or potential interest, in day centres. Many work in health and social care in roles that might involve planning, funding, evaluating and referring or signposting to day centres. Others work in community organizations that might consider partnership working with day centres, or in organizations supporting other stakeholders, work or volunteer in day centres or research service provision. Personal stakeholders include carers of day centre attenders and attenders themselves.

To inform the overall work programme’s longer-term plans, participants were also asked about their views regarding the role of day centres’ role in care pathways and how they may support any preventive function. Findings concerning day centres’ preventive contribution are reported here.

As the study explored people’s perceptions, we employed qualitative methods as these focus on the participant’s, rather than the researcher’s, perspectives.

### Ethical approval

Ethical approval for this project was granted by King’s College London (LRS/DP-21/22–27013).

### Sample and recruitment

Participants were based in four of London’s 12 south London boroughs^1^ that were selected purposively, taking a maximum variation approach (Bryman, 2012; Cresswell, 2013) centring on extent of deprivation, using the Indices of Multiple Deprivation 2019. These calculate deprivation levels for each small area using data about income, employment, education/skills/training, health/disability and living environment deprivation, crime, barriers to housing and services (Ministry of Housing, 2019). Boroughs are ranked from most to least deprived reflecting the extent of deprivation within each borough (which is a collection of small areas). Compared with other south London boroughs, the extent of deprivation across one borough was very low, high in two boroughs and middling in the remaining borough. The population size in each borough ranged from low to high. Two were inner London and two outer London boroughs.

The ten participating day centre participants were recruited by telephone or email, from a sample of 39 older people’s day centres across the four boroughs identified in a previous mapping study (Green et al., 2021). A further eighteen people were invited to participate but did not respond to follow-ups. Contact was not possible with two day centres and two no longer operated. Eleven were not contacted as we had reached our target sample.

Stakeholder recruitment, to a maximum of 10, was purposive, through known networks, for example, by approaching people who have expressed interest in our work, and via calls for people to share their professional perspectives on public internet sites such as X (formerly Twitter) and our study web pages. Nine stakeholders participated, with a further interview scheduled but cancelled. Of the 39 other stakeholders contacted directly, two declined and 37 did not respond.

A recruitment flyer was followed up with an Information Sheet (PIS), information about withdrawal arrangements and the opportunity to ask questions. Pre-interview consent was written or oral and included agreement to roles and LA areas being reported. A Certificate of Participation in Research was offered. Participants in non-professional roles were offered a £20 shopping voucher to acknowledge their contribution.

### Data collection

One-off interviews took place during April and May 2022 with 19 participants, lasting 11 hours and 33 minutes in total. Interviews were online using Teams or Zoom (*n* = 16) or by telephone (*n* = 3), according to participants’ choice, at times convenient for them, and were audio-recorded, with permission.

As an adjunct to a topic guide developed around the priority setting survey findings and planned coverage of the resources, a final question elicited views concerning whether and how day centres contributed to prevention efforts of health and social care services.

### Data analysis

Audio recordings were transcribed verbatim professionally and participants pseudonymized by code allocation. Transcripts were then checked, read several times for familiarization and analysed using a framework approach (Miles et al., 2014). Data on perceptions of day centres’ preventive role were specifically extracted (KO) and analysed using a framework incorporating the three levels of prevention, and strengths and opportunities (KO & KS). Participant roles and LA areas are identified in outputs. Data supporting the findings of this study are not publicly available due to ethical restrictions but are available on request from the corresponding author.

Reporting adheres to the Standards for Reporting Qualitative Research (O’Brien et al., 2014) to demonstrate transparency, authenticity and credibility. The female research team’s backgrounds are in gerontological social care and health research.

### Service user, public and community involvement, engagement, and participation

The programme’s PPIE lead and deputy commented on the findings of the priorities survey, which led to this study, and were requested for feedback on proposals for this study.

## Participant characteristics

Nineteen people from the boroughs of Kingston upon Thames, Lambeth, Lewisham and Merton participated (see Table I). The day centre participants (*n* = 10) were strategic or day-to-day managers/coordinators, and one was a volunteer and service user. Other stakeholders (*n* = 9) were varied and some brought multiple roles or previous experience of different roles to their interviews (see Table II). Day centres were local authority, voluntary sector or commercially operated (see Table III).

**Table I.** Participant breakdown overview.

Participant groups	Kingston	Lambeth	Lewisham	Merton	*Totals*
Day centre participants	2	1	4	3	***10***
Other stakeholders	3	5	0	1	***9***
***Totals***	***5***	***6***	***4***	***4***	***19***

**Table II.** Participants’ roles and identification (ID) codes.

ID code	Participant roles
	**Day centres**
P01	Day Services Manager, Adult Community Services—Disability & Older Persons (employed by LA)
P03	Coordinator (employed by hosting church)
P04	Day Centre Dementia Team Manager (employed by LA)
P05	Day Opportunities Manager (employed by LA)
P06	Service Manager, Adult Social Care—Community Services (covering a range of internally provided services including enablement, Shared Lives, Duty Team, Telecare, Day Opportunities, Specialist Dementia Services)
P07	Care Home and Day Centre Manager
P09	Deputy Chief Executive
P12	Office Manager
P13	General Secretary
P14	Volunteer and service user
	**Other stakeholders**
P02	Social prescriber & former DoLS (Deprivation of Liberty Safeguards) Older People’s Social Worker who gathered team’s views beforehand.
P08	Family carer of former day centre attender and registered social worker
P10	Hospital Occupational Therapist
P11	GP who is involved in local borough-level governance with a portfolio that includes adult mental health and primary care.
P15	Strategic Director Integrated Health and Care, LA & CCG & with oversight of public health and social care
P16	Director of Integrated Commissioning (Adults), LA & CCG, with oversight of Public Health
P17	Acting Director Adult Social Care
P18	Interim Commissioning Lead Learning Disability (leading on day opportunities for all age groups)
P19	Service Development Officer—Learning disability and carer services in Commissioning team. Role includes managing the contract with Alzheimer’s Society; gathered and reported views of Alzheimer’s Society in borough also.

**Table III.** Overview of participating day centres.

Day centre (n = 6)	Sector	Target service users	Number of participants
DC1	Local authority	Older people living with low to moderate dementia	1
DC2	Local authority	Older people living with dementia with high needs	3
DC3	Voluntary sector (paid coordinator, volunteers)	Older people (aged ≥60) including people living with dementia	1
DC4	Private	Older people living with early dementia	1
DC5	Voluntary sector (paid manager and staff, volunteers)	Older people (aged ≥55) fully independent with personal care and able to control the effects of any medication	1
DC6	Voluntary sector (paid manager and coordinator, volunteers)	Older people (aged ≥55)	3

## Findings

**Figure 1.**
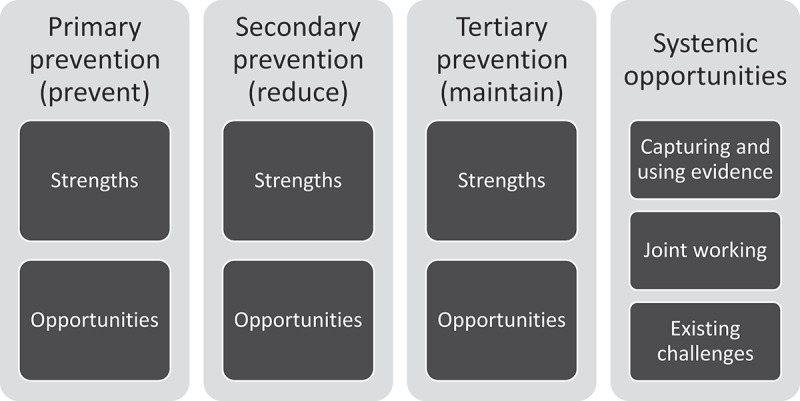
Identified themes and sub-themes.

Findings are presented under four themes (see Figure 1). The first three cover the three levels of prevention, and strengths and opportunities within these. The fourth covers systemic opportunities, including capturing and using evidence, joined-up working and existing challenges; these opportunities can be experienced across the spectrum of preventive goals. Illustrative quotes appear alongside themes.

### Primary prevention (prevent)

#### Strengths

The continuity offered by day centre communities, which builds trust and enables monitoring and subsequent action, is central to the preventive activities undertaken and presents an important opportunity to maximize preventive activity:

It’s the continuity that we get to know people for a longer period of time (…) you get to know the family intimately and their home situation. (…). And because social workers are often, they are, you know, assigned to someone, and then they’re pulled off. (…) But then we are the consistent factor. (…) So yeah, it goes much further than just the singing and clapping. (P04, DC provider)

Participants considered day centres to be communities that maintain health and wellbeing by monitoring their older attenders regularly, preventing their decline and intervening early to prevent crises:

I think it’s the role day centres have as preventative services, in preventing people in declining. (P09, DC provider)

Regular day centre attendance enables early action on safeguarding matters if, for example, a routine chat with an attender’s family member reveals safeguarding concerns:

We also actually contribute to safeguarding because we get to know somebody so intimately. And we suss out, “Hey, there’s a bruise”, “Hey, that carer is by now sounding so distressed”. And they’re talking to us, they slip in their conversation, “and I’m locking her in her bed if I’m at home,” (…) So we would then actually work together with the safeguarding team and say we just heard some concerning things, and that would have then triggered a social work visit. (…) It’s very discreet, things like that, because you need to build up a rapport with the relatives before they feel safe enough to open up. (P04, DC provider)

We do a lot of safeguarding. You know, if somebody needs to come to us because, something’s happened at home. (P01, DC provider)

Some, like one social worker, viewed preventing social isolation, a health risk factor, as day centres’ primary role.

Preventive support for carers was enabled by one day centre which had supported its LA to identify carers who may not be taking up available support; it did this by enclosing information about the LA carer identification project with its regular invoices:

We’ve recently been involved in a project that has been funded by the council to identify carers who are not necessarily unknown to the system, but who may not be taking up available support. One of the ways we decided to communicate with the carers who are on our radar was by sending them out, sending information about that project with their bills. A lot of people are paying their day centre bills by direct debit now, that’s our preferred form of payment. So that means something goes out in the post every month. (P09, DC provider)

Providers perceive their services as also improving the wellbeing of the younger older people who volunteer in them:

The volunteers that we have working in day services are very often older themselves. Not always, but they are. So, we do see that as an important part of our work, we’re providing an opportunity for those people, through volunteering. (P09, DC provider)

This is exemplified by a day centre volunteer whose depression led her to start attending and later volunteer; this improved her mood and motivation, which, she felt, contributed to less frequent use of NHS services:

It has helped me quite a lot. It makes me get up and get out and exercise whereas, before I wasn’t. You know, just like if you’re not going anywhere, you don’t bother getting up and you don’t bother washing or dressing, but because you know you’re going out, you have to make an effort. (P14, DC provider - volunteer & service user)

A belief was expressed about the general understanding of the “trickle-down” effects of this sort of low-level input to people’s wellbeing and by which wider health and care services would also benefit:

When you increase the wellbeing and the happiness of elderly people, it has a trickle-down effect on many things. So I think, indirectly, the NHS will benefit as well, because it may reduce GP attendance. Yes, so I do believe that’s partly affecting the NHS as well. But most day centres are funded partly by the local authority and from different funders, not from the NHS, unless it’s linked with occupational therapists, and I’m not quite sure if that’s the case. But I think even though no funding is going there from the NHS, the NHS is benefiting from it. So, if there could be, I don’t know, other research or other great ideas about – because what happens with social prescribing now, we are referring all those people back into the community. The NHS saves millions and millions of pounds. They were scrutinised for it. And now the NHS is also giving us a capacity fund. It’s a modest amount, but they’re giving us funding to allocate back to the voluntary sector because we were over prescribing some services, just to build capacity. So maybe that’s also possible for day centres to get some funding from the NHS. (P02, social prescriber)

#### Opportunities

Two commissioners were keen for public health activities to take place in day centres:

One model I was thinking about was how you can bring in public health as well, (…) where you can get all types of support, and information, and advice, and vaccines, and health needs, smoking cessation, you know, all those type of things in the same place. (P18, commissioner)

### Secondary prevention (reduce)

#### Strengths

Overall, participants recognized day centres’ preventive role as multi-faceted, covering social inclusion, maintenance of skills, stimulation, early intervention for identified problems or changes, and delaying further deterioration that may lead to an expensive care home move:

We talk about maintaining people, where we can, at the level, but I think it is preventative if your social needs are met. You know that you have, you feel included and I think being part of a service for some people, who maybe live alone, don’t have family, able to stay at home but they’re able to socialise, maybe keep their skills, maybe take part in an activity. We’re looking at activity programmes. So we prevent them needing to go to hospital because of some unknown reason. There may not be anything fundamentally wrong with them, but they need to be checked out because their behaviour’s changed, or they need to go into a home prematurely, when it doesn’t meet their needs properly, you know, and it hasn’t, you know, if we’re talking about finance, it has an expense on everybody. (P05, DC provider)

Supervision in a physically appropriate environment means some people, such as those at risk of falling, being safer than they would be at home, whether they live with others or alone:

But that is sometimes exactly why (…) people would get referred. They are not safe at home. Because the daughter or the son is working and they cannot be left alone (…). So that could be the reason, whether that person wanted to come or not. But that is, it’s either that or go in a care home. So, a day centre is definitely the better option then. (P04, DC provider)

People who come to the day centre most of the time they depend on the carers. Carers only come in once in the morning or once in the evenings. Between time, there is a risk of falls as well. But at least that won’t be there, so in a way, reducing hospital admission to a certain extent - for like unnecessary hospital admissions. (P07, DC provider)

Several participants explained how day centres prevent Urinary Tract Infections (UTIs) worsening because they provide personal care or notice an attender may be confused. Noticing a UTI early on—and other monitoring activity described in this article that leads to early intervention—can be particularly beneficial for people without family or who live alone, especially when a day centre is able to fast-track someone into the GP system:

*[Talking about how the* *day centre records activities such as calling the GP to make an appointment for a* *person whose urine may be smelly and staff suspect a* *UTI]* Very often it will be us who, we’ll be the first people who see that person and might identify that something, you know, it might be their behaviour - that they are confused when they’re not normally confused. We might see that before anybody else, if that somebody is living on their own. (P09, DC provider)

*[Talking about one of the main reason for people with dementia being admitted to hospital being a* *UTI]* So, if there’s somebody coming into the day centre, their health is being monitored throughout the daytime, so it’s less likely they will develop some kinds of things. And if they do, they will have something that is easy to tackle with the GP. (P07, DC provider)

The service’s role was likened to a substitute social work service that supports care partners and prevents crises:

I would argue that we became, like, sometimes, a substitute social worker, in the end. The person with dementia presents relatively straightforward (…).But it turns out that their family member, they’re on the phone every day, and we’re like, Oh, yeah, you know, that person is phoning again. It’s not going to be a five-minute call. It’s going to be half an hour because, actually, they’re the ones with mental health, or they are so under pressure, or they are so struck by where they have multiple other concerns in their family life. So, we become a safety net for them. (…) and we are preventing some sort of service somewhere down the line from serious trouble because we are the ones absorbing that. (P04, DC provider)

Day centre participants detailed the services’ varied role in supporting older people’s care partners through respite provision, a listening ear and enabling them to access support. Services can also potentially delay a permanent care home move resulting from carer crisis, and also act as a stepping-stone that makes the eventual transition less stressful:

Then family doesn’t have to think about moving their loved one into a care home. In the beginning, they can, as an initial step to the – before going to the care home. So, in a way, even if social services is funding, then funding only needs to start at like maybe six months or maybe a year later, unless, you know, the symptoms get worse. (…) They’re taking a new step going to the day centre once a week or twice a week or three times a week. Then eventually become a respite for a couple of weeks, then become residents in the care home. So they don’t really feel overwhelmed. (P07, DC provider)

#### Opportunities

Several current and missed opportunities for secondary prevention were highlighted. A voluntary sector provider was involved, with its local Primary Care Network (PCN), in a proactive, anticipatory care pilot that targeted older, frail people using an MDT approach; an expected benefit would be increased referrals to the centre:

It’s about identifying people who have rising needs and using an MDT approach to make sure that they are supported more effectively and have got a care plan in place. So again, that’s also a source of referrals into the day service, and that’s a requirement for anticipatory care is in the new GP contract (…). So we’re really pleased that were involved in that, upstream. (P09, DC provider)

The GP mused the potential for a degree of shared medical monitoring—which would need to be planned properly and undertaken safely, with relevant information about individuals being made available—but felt that simple referrals would be best:

I think what would be useful would be just the ability to kind of communicate concerns and so that, if a centre is concerned that somebody is unwell, you know, they would know who the GP is and could send a message saying, This is why you might wish to call this person and review them, depending on your level of concern. (…)

We are already moving a lot of blood pressure monitoring nationally and locally, letting people do it themselves at home. And if someone is not able to do it themselves, then obviously a worker or a carer could do some of that on their behalf. So, I think it’s certainly worth giving thought to, but I think it raises some expectations about how it is done well and reliably and where accountability lies and different things like that. So that, you know, sort of bad examples of where someone monitors and writes a lovely sort of detailed thing in a book every day, but, actually, doesn’t know what the threshold is to be worried, and they just simply carry on writing something in a book every day, that’s a bad outcome. It’s a waste of time, but also potentially dangerous. But, so, I think if we’re talking about something that actually starts to be shared care, effectively, then I think there are expectations to have it done well and safely, and that’s entirely doable, but I think it is a significant step from where we are at the moment, by and large. (P11, GP)

Falls prevention work was highlighted by a commissioner. The occupational therapist highlighted how useful it would be for day centres to provide falls prevention exercise classes, particularly evidence-based ones, such as Otago programmes, and to measure pre-and post-class falls risk or balance scores, and offer toenail cutting:

I would hope that day centres have some kind of exercise programme because, especially with older people, falls are such a big issue. So, I guess if you were, as part of your day centre, you were implementing an Otago type exercise class, which would be really useful, as an OT, in preventing falls for people, and I guess it would be good to measure their pre and post sort of falls risk or balance. There are a few very short kind of physio measures. You can do like a 180 test where you see how many steps, it takes someone to turn around or there’s, the Stand Up and Go. (…) It’s a shame they don’t do the toenail cutting thing. They could do with that. (…) I think people get so scared off, they just don’t touch toenails. It’s too complicated. (P10, OT)

### Tertiary prevention (maintain)

#### Strengths

Providers highlighted how day centres monitoring people after hospital discharge prevented hospital re-admissions. One centre often received referrals from hospital discharge teams:

But if they’re being discharged and they do need a level of observation, we, you know, we’re asked to do that as well. This person really needs to be observed, you know. Can you? (P01, DC provider)

The occupational therapist considered that day centres supported the NHS by maintaining people’s cognitive and physical abilities, finding it very reassuring that people recently discharged from hospital could be referred to a day centre so that an eye is kept on them:

That’s the trouble, you send people home [from hospital] and you sort of you get no idea how they get on, really. So, if you knew that they were being seen once a week or something in a day centre, that’d be quite reassuring. (…) I feel they would pick up things like, you know, a UTI or … ? (P10, OT)

Preventive impact is sometimes clearcut, as demonstrated by this example in which staff identified the causes of a service user with dementia and diabetes becoming unresponsive which led to fewer ambulance call-outs:

We constantly had to phone out the ambulance, but we worked out what it was and how we could avoid it. So therefore, we didn’t have to phone the ambulance all the time as much anymore, you see. (…) I would argue that’s a direct benefit and it evidences the quality of the day service or its existence, because it saved the NHS money. (P04, DC provider)

Day centres were considered well-placed, safe spaces in which new ideas or activities related maintenance of health or independence can be introduced and from which connections can be made to specialist health services that may be hard to access or which people worry about accessing.

Day centres can be a great place to introduce new ideas to people where they are feeling safe. Like Mum really didn’t mind when the dementia nurse came round (…) A lovely nurse could come along and just have some kind of session: “We’ve got [nurse] here now, looking at your health. How can we keep healthy? I know you do exercise classes, but what about brain health? Maybe we might forget that odd word, who’s struggling with it? Here are some ideas [nurse] is going to tell you about.” So, there’s something about finding that optimum opportunity, and they’ve got a few mates with them, it’s not so threatening to talk to the nurse. (…) And if they say, “Well, I’m struggling a bit.” “Well, if you’re happy, I could take your name and maybe, you know, I could follow that up for you.” (P08, family carer and social worker)

For a commissioner, the respite afforded by day centres supports carer wellbeing and reduces potential later use of services, for example:

For me, it’s the prevention around carers (…) respite which supports their wellbeing, which means they’re not accessing support further down the line (P19, commissioner)

#### Opportunities

The long-term nature of day centres’ preventive input was emphasized and how, if better recognized, this could generate further funding possibilities. Although long-term attendance may lead to difficulties attracting new attenders, it also means supporting people for as long as possible, until their decline prevents them from attending:

A lot of the time, once people are referred into the day service, they’re there for years and they may deteriorate. And we did have a period where, you know, can people move on? Where do they move on to? You know, it just means a higher care package; there’s still a cost. (…) we’re stuck in terms of the people that we’ve got, in the nicest possible way, you know? (P06, DC provider)

Specialised day centres for specific cultural or language-speaking groups of people may also present useful opportunities for health outreach and to address taboo subjects:

… and also, you know, for different cultural day services where people might be fearful of accessing for stigma or not understanding. So, having outreach services, where practitioners perhaps, you know, can speak that particular language, or I think, that’s really essential. (P08, family carer and social worker)

A commissioner welcomed the idea of medication provision and management being made available.

A participant explained how dementia nurses do outreach around memory problems or brain health which might lead to referrals directly into dementia services, how talks may lead to Careline referrals (emergency support service), or talks by OTs or physiotherapists could break down taboos around asking certain questions:

Doing an introductory workshop opens the door for referrals from the day services direct to the NHS (…).There are little workshops that OTs and physios could do. (…) So it’s finding out all that and, you know, kind of trying to take the taboo out of it, really, if it’s done in the right way. I think there is definitely a missed opportunity for more joined up working around that and approaching outreach. (P08, family carer and social worker)

### Systemic opportunities

#### Capturing and using evidence

There was recognition that “*having an effective evidence base is something that is really important*” (P16, Director of Integrated Commissioning (Adults), LA & CCG) and acknowledgement that day centres’ preventive input is not always straightforward to evidence and that evidence may be anecdotal. While anecdotes are not universally welcomed, one commissioner highlighted how “soft” outcomes, such as case study stories, bring alive a service’s value by demonstrating its impact, which helps unlock funding:

It helps us with getting more funding. It helps us to share messages to elected members, to seniors. It shows the value of the service in a really succinct and person-centred way. We can share that we can support 500 people with £100,000. We can share that there’s 50 sessions a week. We can share all that quantitative stuff, but it’s the qualitative stuff that actually makes a difference, I think, in people’s hearts and minds. (P18, commissioner)

Individual stories could also “*support the social workers to trust the service*” (P19, commissioner).

Local authority participants opined that *“self-reported wellbeing measures are as important as clinical outcome measures”* (P15, Strategic Director Integrated Health and Care) but also considered that *“outcome-based data around how long it might prevent the need for other care or admission into residential nursing homes would be really useful”* (P17, Acting Director Adult Social Care) and that it *“might also be the activities within it as well, and the choices you might have”* (P15, Strategic Director Integrated Health and Care) whose value could be measured.

The difficulties of recording such preventive activities or input on a larger scale in a way that is impactful were noted; this was felt to impact negatively on funding opportunities:

I think day centres actually provide a huge amount of resources around keeping people out of hospital and, from requiring more medical intervention (…) but it is difficult to record. (P03, DC provider)

The lack of a systematic approach to outcomes data gathering was noted:

In terms of measuring actual outcomes on people’s health, mobility, kind of mental awareness, anecdotally what’s reported to us and what we see in people is that they actually benefit from being at a day centre and socialising and doing activities, but actually a systemised way of kind of gathering that, monitoring that and therefore giving you an evidence base to continue or expand your day centres, I think all local authorities and kind of CCGs struggle with a bit. (P17, Acting Director Adult Social Care)

Although individual outcomes data were felt to be useful, local authorities often had “*reams of data that we don’t then usefully analyse or utilise*” (P16, Director of Integrated Commissioning).

Two providers kept detailed records but had not used these to evidence their impact. One used handover devices to record, for example, GP appointments made for service users—exactly the type of data one LA participant felt would support investment in day centres. The other provider recorded, for example, length of service use and felt they needed support to analyse the data to evidence impact:

We want to be able to analyse that data properly, more effectively, and see what it’s telling us. (…) Instinctively we feel that people who are using our services, our day services are happier, more secure, more supported, have fewer visits, unplanned visits to A&E, and a more appropriate use of primary care, than other people who don’t. We can’t prove that. (…) if we could prove it, it would be very powerful. (P09, DC provider)

One provider raised the importance of updating the evidence about day centres:

There is already some good evidence out there about day services, but it’s probably quite dated now. You’ve got to keep these things fresh and keep redoing them, haven’t you, and making sure that if services are not seen as relevant anymore - even if they are valued by the people who use them. (P09, DC provider)

Another commented that they thought the LA recognized the value of their preventive work, while the NHS does not, but they had never been asked how much money their work saves for the NHS:

Nobody’s ever said to me, “because you’re keeping Mrs Blogs in the day centre four days a week, you’re avoiding National Health treatments; how much is that, if there could be a monetary figure? (…) because you’ve kept this person out of hospital and out of social services, major services, how much does that save?” (…) [Name], for instance, is an exercise teacher and she deals with one class, not in this building, but the average age is in the mid-eighties, and their recovery from, say, knee operations or hip operations is very good. Very often they’ll go to physio for recovering. They say, you know this, you can go. How much does that save? I think it’s very important because I don’t think they recognise it. I think the council do but the NHS doesn’t. (P13, DC provider)

Evidence should not overlook day centre staff and volunteers. One provider, for an application, was required to “*work out the value for our volunteers and for the in-kind hours that are provided, the voluntary hours etc.”* (P03, DC provider). Two commissioners expressed interest in knowing “*how staff felt about working there, whether they felt supported”* (P18, commissioner) and how organisations “took care of their staff wellbeing” as part of the social value offered by a service.

Finally, in measuring outcomes, it’s important to understand that “*personalisation doesn’t have to be the enemy*” (P16, Director of Integrated Commissioning).

#### Joint working

There is room for more joint working with statutory services. An LA day centre manager commented how useful it was to be part of an in-house MDT of managers within the council, which included NHS representatives. They felt this raised awareness of their service and made it easier to encourage staff to increase their knowledge and skills through secondments or shadowing placements:

… heads of service, all the managers across the social work teams, across the hospital teams. And I’ve just lent one of my workers to the hospital team because I want her to sort of beef up her skills (…) So I encourage people to do shadowing in other departments… (P01, DC provider)

One centre received NHS funding for post-discharge support and for preventing hospital admissions, demonstrating some local statutory recognition of day centres’ preventive monitoring role:

They give us money for all sorts of stuff, for hospital discharge, or, you know, to flow, to make it easier for people to come out of hospital, and to stop them going in. (P06, DC provider)

Day centre staff, who see attenders more regularly than social workers, might also be involved in individual reviews:

… feedback from our day opportunities is that members of staff, they’d like to be included in that review because they are such an important part of someone’s whole life really. (P19, commissioner)

They [day centre staff] often know way more about the person than the social worker does (…). These guys see them every week. (P18, commisioner)

Knowing that a patient attends day services, and how often, would provide a useful fuller picture of support received. While some could search for this information on LA databases, administrative boundaries complicate information sharing:

We [GPs] deal with a model of where people are registered. Council services deal with where people live. And these don’t always overlap, so we have sort of cross borough issues. So, a few of my patients live in [different borough] because people don’t neatly align themselves. (P11, GP)

#### Existing challenges

For providers, making organizational links with the NHS was not necessarily straightforward, and it tended to be personal links that led to collaboration. One provider explained how personal health links with a hospital department led to them setting up an osteoporosis group:

I don’t know [what would help GPs know to make referrals to them]. We have tried writing, tried sending leaflets. We always send our handbooks to all the GPs, but I suppose I’m never quite convinced they get to the GP. I have hand-delivered to my own GPs while I’m actually being seen. We do have with places, like the osteoporosis department and the rheumatology department at [hospital], we have a good relationship with because I actually attend them and they’ve asked me to set up an osteoporosis group in [town] because there isn’t one. (…) So we’ve got links like that, but they’re very, very personal links. (P12, DC provider)

## Discussion

These findings provide important insights into perceptions of how day centres’ work can support through monitoring activities, the preventive priorities and agenda of health and care services. They reveal recognition of day centres’ preventive work at different levels and prevention-related strengths and opportunities, and confirm that day centres are aware of their own preventive monitoring strengths.

Findings highlight work undertaken in day centres that falls under the radar as a systemic contribution. For example, monitoring conversations exemplify the type of “health and wellbeing conversations” taking place in third-sector organizations that contribute to making every contact count (Nichol et al., 2024). Health and wellbeing monitoring in older people’s day centres aligns with NHS efforts to move care upstream—away from health to community settings (NHS, 2019) and has been likened to “added” value for systems since it does not fall within the reasons for which centres may have been funded or individual places purchased (Orellana et al., 2020a). Added value is a marketing concept referring to something that supports choices about what to purchase (Sheth et al., 1991). Day centre services and individual places may be purchased by individuals (self-funders or direct payment holders), statutory bodies or other funders.

We concur with other recent English research concluding that day centres, as trusted places that provide collective care and have a variety of strong local links, could play a more active role within local preventive initiatives and Integrated Care Systems (Bennett et al., 2023; A. M. Cameron et al., 2024). We now discuss points relating to day centres’ contribution to the provision of anticipatory care and a case for more widespread joint commissioning of day centre places.

Preventive work undertaken in day centres, facilitated by their person-centred, relational nature, can be linked with the anticipatory care agenda. Anticipatory care is proactive healthcare and support targeted at people living with frailty, multiple long-term conditions and/or complex needs to help them stay independent and healthy for as long as possible in their own home. It is a contractual requirement as of 2023/24, with Primary Care Networks making plans and anticipatory care services themselves being led by Integrated Care Systems (ICS). ICSs are partnerships of organizations that come together to plan and deliver joined up health and care services and to improve the lives of people who live and work in their area and deliver better value for money. English research reports that half to two-thirds of day centre attenders live alone and a high proportion have multiple long-term conditions (Lunt et al., 2021; Orellana et al., 2020a). Analysis of one South East London GP surgery’s records showed that, compared with those who co-resided, people aged 65 and older living alone were much more likely to attend A&E, were at increased risk of being admitted to hospital as an inpatient, were more likely to have significantly more GP appointments, and a higher proportion had more long-term conditions (Dreyer et al., 2018). Rather than focusing solely on those with the greatest needs, policy analysis suggests that some demands on health and social care services could be alleviated by shifting some preventive, upstream attention to older people with early frailty (Drennan et al., 2018). Day centre attenders are, therefore, a population of interest with respect to anticipatory care. The strong relationships in day centres and with carers—that staff and volunteers enjoy developing (Orellana et al., 2021)—are a strength that enables responsiveness (Bennett et al., 2023; Cameron et al., 2024).

Joint commissioning (integrated health and social care commissioning) is becoming increasingly important health and social care in England. The introduction of ICSs brings hope of increased joint commissioning; study participants were keen on increasing joined-up working with statutory services. However, new initiatives can take a long time to settle in and there are inherent challenges to working across social care and health. For example, twenty years after budget pooling was allowed to enable joint commissioning between the NHS and LAs from April 2000 (HM Government, 1999) little prevention work was being undertaken collaboratively between adult social care and health (Marczak et al., 2019). Contributing factors include differing conceptualizations of “prevention” (e.g., preventing decline versus cost-saving), different priorities, cultures and information/technical systems (Marczak et al., 2019). Further detail of the complexities around the concept of prevention are discussed by Verity et al. (2022). Overall, there is a lack of evidence about outcomes of combined health and social care work (Dawson et al., 2020) and about preventive social care services’ effectiveness and their cost-effectiveness generally (Marczak et al., 2019). Analysis of joint commissioning discourses reveal a link between preventive commissioning efforts and efficiencies (i.e., cost-savings) and a focus more on people with the greatest need who incur higher costs than people with lower needs (such as day centre attenders), and it is merely an assumption that joint commissioning will deliver efficiency savings at the level expected (A. Cameron et al., 2018).

Value placed on evidence type is a further barrier to joint commissioning. Because procurement often requires economic evidence, qualitative evidence may be considered insufficiently robust to justify investment by both social care and health (Marczak et al., 2024). It will be important to develop understandings of how rigour may be demonstrated in qualitative research (Davies & Dodd, 2002) although time constraints may present challenges here. In adult social care, opportunities, and skills needed, to analyse and reflect are lacking (Marczak et al., 2024) and this is likely also the case in NHS decision-making settings.

Furthermore, there appears to be a prevailing tendency to focus on commissioning innovation rather than maximizing existing services’ impact. Interestingly, professional stakeholders in our study were more focused on prevention-related development opportunities than officializing or maximizing centres’ current preventive monitoring role. LA commissioners have previously raised how using day centres as outreach venues to reach larger groups of people is attractive, but also the reluctance to pay for building use even in the more integrated councils (Orellana, 2018).

Ever-changing NHS structures and a fragmented NHS (Melin Emilsson et al., 2022) and frequent internal reshuffles in LAs present additional difficulties. Study participants echoed earlier findings that developing organizational relationships with part of the health system were challenging (Orellana, 2018). Not only are these difficult to establish, but constant change threatens those made. With change comes both fresh knowledge and expertise and lost knowledge and expertise about local services and their (potential) benefits.

There is national and international evidence that day centres help maintain or improve their attenders’ quality of life, support family carers and provide wellbeing benefits for volunteers whether through attendance alone or participation in preventive interventions as part of day centre services (Bennett et al., 2023; Du Preez et al., 2018; Ellen et al., 2017; Hagan, 2015; Honjo et al., 2020; Lunt et al., 2021; Maffioletti et al., 2019; Marquet et al., 2020; Naruse et al., 2020; Orellana et al., 2020a, 2020b, 2021; Österholm et al., 2023; Parker et al., 2021; Rokstad et al., 2019; Siette et al., 2022; Strandenæs et al., 2018, 2021). The diversity of provision and funding, care and health systems within which day centres operate is important to acknowledge when considering the potential for day centres to contribute more formally to preventive agendas. For example, it has been noted that the research reports more preventive interventions taking place in day centres outside the United Kingdom in countries where, perhaps, day centres are a more integral part of the health and social care pathway (Orellana et al., 2020b). In England, improved recording of preventive-related work may support the shift towards a more recognized “role” for day centres, both within the social care service eco-system (Burn & Needham, 2023), and more broadly, within social care and NIHS prevention work.

### Implications of findings

Recognition and investment are needed to maximize day centres’ upstream preventive contribution. Day centre financing is notoriously unstable and complicated, and these services would like to develop their sustainability for the future (Orellana et al., 2024). Three factors combine to present a case for improved joint LA and NHS commissioning via Integrated Care Systems, and for individual day centre places to be commissioned by GPs where this identified as appropriate and patients are agreeable. LAs’ responsibility to ensure a choice of high-quality care services is available for people who need them (HM Government, 2014), a local requirement for anticipatory care, and the preventive added value day centres can offer to the NHS. Policymakers and statutory decision-makers need to take individual responsibility for ensuring they are aware of the extant evidence about day centres’ outcomes. GPs are in a position to identify people who may benefit from such non-clinical community support (Dreyer et al., 2018) and may wish to develop links with day centre providers.

Examining the content of commissioning, procurement, social prescriber and medical education would contribute to understanding whether content could be developed to cover the evidence base around these commonly commissioned or used services.

Providers and their stakeholders would like more evidence about day centres’ systemic and financial benefits (Orellana et al., 2024). Our findings support previous calls for research about potential health and social care savings linked with their use, both in England and elsewhere (Dabelko & Zimmerman, 2008; Orellana et al., 2020a). There is a need to map day centres’ monitoring activities and processes and any impact monitoring has on different elements of the health and care systems, including on staff workloads, together with any outcomes for older attenders themselves.

### Strengths and limitations

Strengths lie in information-rich study participants and wide range of professional and practitioner roles in four very different south London boroughs. Despite the small sample, these findings can be regarded as representative of a larger group of day centres and their stakeholders, as participants articulated similar views to previous unpublished research (Orellana, 2018). However, study participants were already aware of day centres which are, perhaps, not representative of the broader group of professionals in similar roles. Given that a single question was asked on this topic, participants outlined their views only briefly; further questions may have enabled them to provide additional information that would have further enriched these findings.

## Conclusion

Day centres for older people are perceived to be well-placed to contribute more formally to the prevailing preventive agenda of health and care services. Their activities to monitor attenders, intervene when needed, and maintain well-being align with the primary, secondary and tertiary prevention framework. However, tight budgets and current limited joint commissioning practices potentially miss the added value of these activities, and the benefits of anticipatory care day centres may offer. It is, therefore, important to ascertain more clearly what this contribution is and its financial worth.

## Note

1. Bexley, Bromley, Croydon, Greenwich, Kingston-upon-Thames, Lambeth, Lewisham, Merton, Richmond, Southwark, Sutton, Wandsworth.
